# Medical and Philosophical Intelligence

**Published:** 1812-03

**Authors:** 


### MEDICAL AND PHILOSOPHICAL INTELLIGENCE.

### ROYAL SOCIETY.

JAN. 9*th and* 16*th*.—The conclusion of Dr. Herschel's paper on the late comet, entered into a very minute investigation of the nature and extent of the luminous matter which surrounded it at some distance from the planetary body in its centre. This matter the doctor supposes to be of a phosphoric nature. The length of the tail he estimates to be, at the end of October, about 100 millions of miles; but to be very variable in length and breadth, and a hollow cone, emitting light on all sides. The inner side he supposes may illumine the planetary body in a manner similar to that in which the ring does Saturn. From the great alterations which took place in the nature and dimensions of the tail, he is inclined to conjecture that comets may be formed of nebulæ; that those nebulæ undergo condensation in their approach to the sun, or to some of what are called the fixed stars; and that in process of time they may become regular planets. On contrasting the appearance ance of the late comet with that of 1807, he is inclined to suppose that of 1811 much younger than the former.

*Jan. 23d.*—Mr. Davy communicated a paper written by Mr. Campion, on the structure of the eye of man and birds, particularly in relation to that faculty which enables it to adjust its focus to the distance of the object. The author examines the different conjectures and theories which have been proposed to account for this circumstance, and explains how the eye can have perfect images of objects at very different distances. It has been generally agreed that the eye must have some contractile power; but the existence of any organ capable of such a function, has never been ascertained. Mr. C., on examining the eye of an eagle, discovered the existence of a small muscle attached to the sclerotica, and capable of contracting the eye, in a manner equal to effect the necessary change in the focal distance. The same muscle he discovered in some other birds, and hence he inferred that something analogous exists in the human eye. He observes, that images pass before the eyes of maniacs as vividly and distinctly without any sensible objects, as they do over those of some persons from objects within the focal distance of their eyes.

---

Mr. Jesse Foot has in the press, a Critical Examination and Review at large of Mr. Everard Home's "Observations on the Treatment of the Diseases of the Prostate Gland."

---

Dr. De Lys, of Birmingham, has in the press a Translation of Richerand's Elements of Physiology, from the fifth and last edition. This work will be illustrated with notes by the translator, and accompanied by a comparative view of the state of physiology in this country and on the continent. It will form one volume 8vo. and is in great forwardness.

---

Staff-Surgeon Thomas, who is just arrived from his station in Portugal, has nearly ready for the press, a Statement of the Condition of the Medical Military Hospitals of the Portuguese, from the time the British staff were originally appointed to act under Marshal Beresford, to the present period.

---

*Scientific Institution, No. 3, Prince's-street, Cavendish-square.*—Mr. Singer will commence a course of Lectures on Chemistry, embracing a popular exposition of the most interesting discoveries, on Tuesday the 3d of March, at eight o'clock in the evening, and continue them on each succeeding Tuesday.

*Arrangement of the first Six Lectures.*

Tuesday, March 3d.—Lecture 1st.—Nature of chemical inquiry. Its objects and application. Chemical distinguished from mechanical action. Powers by which the phenomena of chemistry are produced. Attraction. Experimental illustrations of this power.

Tuesday, March 10th.—Lecture 2d.—Powers productive of chemical phenomena. Heat, or caloric. Its different states of existence, tence, propagation, and effects, illustrated by experiments. Economical and useful application of these principles.

Tuesday, March 17th.—Lecture 3d.—Properties and effects of heat continued. Influence of heat on the form of bodies. Professor Leslie's experiment on the congelation of water by evaporation. Formation and nature of gases, or different species of air, illustrated by numerous experiments.

Tuesday, March 24th.—Lecture 4th.—Chemical composition of the atmosphere. Properties and proportion of its constituents. State of the air in ball rooms and theatres. General property of oxygen and nitrogen gases. Their relation to natural phenomena.

Tuesday, March 31st.—Lecture 5th.—Hydrogen or inflammable air. Its sources and effects. Combination of hydrogen with oxygen. Composition of water, shewn by analysis and synthesis.

Tuesday, April 6th.—Lecture 6th. Of simple or undecomposed substances, and the experiments that have been made to ascertain their nature. Inflammables. Sulphur. Charcoal. Phosphorus. Experiments illustrating the properties of these bodies.

---

*Westminster Lying-in Institution, Queen-square.*—A course of Lectures will be given, during which each pupil is to deliver one patient a week for illustration. They commence the 7th instant, by J. Hopkins, Surgeon to H. R. H. the Duke of Kent, and W. Pearson, Surgeon of the Royal College.

